# Evaluation of Surgical Site Infection in Mini-invasive Urological Surgery

**DOI:** 10.1515/med-2019-0081

**Published:** 2019-09-15

**Authors:** Jacopo Adolfo Rossi de Vermandois, Giovanni Cochetti, Michele Del Zingaro, Alberto Santoro, Mattia Panciarola, Andrea Boni, Matteo Marsico, Gianluca Gaudio, Alessio Paladini, Paolo Guiggi, Roberto Cirocchi, Ettore Mearini

**Affiliations:** 1 University of Perugia, Perugia, Outside U.S./Canada Italy; 2 University of Perugia, Dept. of Surgical and Biomedical Sciences, Urology clinic, Perugia, Italy; 3 Department of General Surgery and Surgical Specialties "Paride Stefanini";, Sapienza University of Rome, Rome, Italy; 4 Department of Digestive Surgery and Liver Unit, University of Perugia, Perugia, Italy

**Keywords:** Minimally invasive, Urology, infection, Surgery, Surgical Site, Hospital-acquired infection

## Abstract

Surgical Site Infection (SSI) is the most frequent source of infection in surgical patients and the second most frequent cause of hospital-acquired infection. The primary aim of this prospective study was to compare SSI occurrences between minimally invasive surgery (MIS) and open urological surgery. Secondly, perioperative outcomes were evaluated in two different approaches.

A consecutive group of 60 patients undergoing urological surgery were prospectively enrolled in a single high-volume center between May and October 2018. We included procedures that were performed by minimally invasive or traditional techniques. We evaluated and compared the incidence of SSI and perioperative outcomes in terms of intraoperative bleeding, post-operative complications, postoperative pain, patient satisfaction with the analgesic treatment, time to flatus, time of oral intake and mobilization, and length of hospital stay. The two groups were homogeneous with regard to demographic data.

Superficial incisional SSIs were diagnosed in 10% of cases (3/30) in the second group and 0% in the first (p<0.05); space/organ SSIs developed in 4 patients, which were diagnosed by ultrasound scan and confirmed by abdominal CT: 1 patient (3.3%) in group 1 showed an infected lymphocele, whereas 1 case of infected lymphocele and 2 cases of pelvic abscess were detected in group 2 (10%, p<0.05). All the perioperative outcomes as well as were overall complication rate favored MIS (p<0.05). The use of minimally invasive techniques in urological surgery reduced the risk of SSI by comparison with a traditional approach. In addition, MIS was associated with better perioperative outcomes and a lower overall complication rate.

## 1 Introduction

Postoperative infections are a common complication and cause of morbidity. According to the Centers for Disease Control and Prevention (CDC) National Nosocomial Infections Surveillance system, SSI is the most frequent source of infection in surgical patients and the second most frequent cause of hospital-acquired infection [1, 2, 3].

In the United States, every year about 500,000 patients develop an SSI. Physical discomfort of the wound, altered cosmesis, delayed recovery, and consequently longer hospitalization and increased costs of care are just some of the sequelae of SSI. Indeed, patients with SSI are 60% more likely to be admitted in an intensive care unit, 5 times more likely to be readmitted, and more likely to have twice the incidence of 30-day mortality than surgical patients without SSI [4]. In addition to preventive measures to reduce the SSI risk during the perioperative period, the type of surgical approach may affect the risk for SSIs [5]. Since its introduction in world surgical practice, the laparoscopy and ever more frequently Robot-Assisted Surgery (RAS) have been used successfully in several urological procedures; their safety and efficacy have been demonstrated. The main indications for Minimally Invasive Surgery (MIS) in urology include radical prostatectomy, radical and partial nephrectomy, radical cystectomy, pyeloplasty, adrenalectomy, colposacropexy even in cases where, because of increasing spread and success, the surgical indications will be extended [6,7]. RAS maintains the benefits of laparoscopy with additional advantages, including magnification of the operative field due to three-dimensional vision, and greater accuracy in dissection and suturing due to EndoWrist® instruments (Intuitive’s multi-functional da Vinci instruments) with seven degrees of motion, primary surgeon camera and control, tremor filtration. The main disadvantages of this system are the lack of haptic feedback and the high costs; however, this technology allows reproduction of the same surgical steps of open surgery with the benefits of minimally invasive technique, which overcomes the limitations of the laparoscopy: in particular, RAS reduces physical strain and simplifies the operator’s learning curve. Moreover, MIS ensures positive perioperative, oncological, and functional outcomes; for perioperative outcomes, RAS seems to reduce intraoperative blood loss and transfusion rates, duration of catheterization when bladder-urethral anastomosis was performed, length of hospitalization, and readmission rates with respect to laparoscopy. It is estimated that SSIs involve about 5% of all surgical patients, but this rate may increase in procedures including opening of the gastrointestinal or urinary tract. Indeed, the incidence of SSIs after radical prostatectomy was reported to be between 0,9% and 16,1%. Although some studies showed a decrease of SSIs with MIS, others highlighted no difference in SSI risk between two different approaches [8, 9, 10, 11, 12]. Regardless, most of these studies are biased by their retrospective nature and have many other limitations [13].

To our knowledge, there is no relevant prospective study published to date that compared the incidence of SSIs after urological MIS and urological open surgery, including in the two groups different types of urological interventions [14,15]. The primary aim of this prospective study was to compare the occurrence rates of SSI between MIS (laparoscopic and robotic) and open urological surgery. Secondly, perioperative outcomes (intraoperative bleeding, post-operative complications, postoperative pain, patient’s satisfaction with the analgesic treatment, time to flatus, time of oral intake and mobilization, length of hospital stay) were also evaluated in two groups.

## 2 Materials and methods

After institutional review board approval was obtained, 60 consecutive patients undergoing urological surgery were prospectively enrolled in a single high-volume center between May and October 2018. We included 4 urological procedures that were performed by minimally invasive (laparoscopic or robotic) or traditional techniques (radical prostatectomy, radical cystectomy, partial nephrectomy, nephroureterectomy) and selected based on the following inclusion criteria: 1) the procedure is performed frequently and represents a urological topic; 2) the minimally invasive approach for the procedure has been in use for at least 5 years; 3) the open approach should not be reserved to more complex cases. The urinalysis and urine culture were performed 3 days before surgery for all patients to ascertain absence of urinary infection. Urinary tract infection was the only exclusion criteria. All the patients underwent a standardized antimicrobial preparation before surgery with povidone-iodine and antibiotic therapy with piperacillin/tazobactam 4.5 g within 1 hour before surgical incision and continued up to the seventh postoperative day. The study population was divided into two groups: group 1 included a series of 30 patients undergoing laparoscopic or robotic surgery; a series of patients undergoing open surgery were included in group 2. In both groups we evaluated and compared the incidence of SSI and perioperative outcomes in terms of intraoperative bleeding, post-operative complications, postoperative pain, patient’s satisfaction with the analgesic treatment, time to flatus, time of oral intake and mobilization, length of hospital stay. Specifically, we used the “Assessment of Post-operative Pain and of the Degree of Satisfaction of the Patient” Questionnaire in order to assess post-operative pain as well as the patient’s satisfaction with the analgesic treatment received. This validated questionnaire was administered to each patient and managed directly by the interviewer who completed the compilation. The questionnaire was administered on the first and third postoperative day and at the time of discharge; it consisted of 11 questions grouped into three sections:

– Structural variables, which included information such as: name, surname, gender, age, marital status, educational qualification, work activity, date, and type of surgery (questions 1-9);
– Pain level perceived, based on the Numeric Rating Scale (NRS) (question 10);
– Satisfaction with post-operative pain management (question 11).

As the study population was characterized by a fairly high mean age, we chose one-dimensional evaluation scale, the Numeric Rating Scale, to evaluate postoperative pain (Table 2.1). The patient was asked to indicate, using a number: the intensity of the pain perceived on a numerical scale ranged from 0 to 10: the number zero indicated the absence of pain, whereas 10 corresponded to the maximum degree of pain. If the value of the pain was between 0 and 3 and the clinical parameters were normal, the physician continued to administer the set therapy until the new control, after 1 to 2 hours. A value of 4, on the other hand, corresponded to the threshold value over which the specific pharmacological therapy had to be set or modified. The perioperative complications were evaluated by Clavien-Dindo Classification. All data were stratified according to the surgical approach and compared using the Fisher test and Chi square test for the nominal variables, with the *t*-test and Mann-Whitney test for the continuous variables. For statistical analysis, the software SPSS ver. 21 (SPSS Inc., Chicago, IL, USA) was used, designating a significance cut-off value P <0.05.

**Ethical approval**: The research related to human use has been complied with all the relevant national regulations, institutional policies and in accordance the tenets of the Helsinki Declaration, and has been approved by the authors' institutional review board of Perugia University.

## 3 Results

The two groups were homogeneous with regard to demographic data (table 1). There were no statistically significant differences regarding sex, age and BMI (p> 0.05). Because of the small number of females in both subgroups, a potential correlation between sex and risk of infections could not be evaluated. Regarding the educational qualifications, out of 30 patients 13 (43,4%) had gained the Elementary School License, 12 (40%) the Junior High School License, 4 (13.3%) were graduate and 1 had a Degree (3.3%). In the second group, 18 (60%) had gained the Elementary License, 6 (20%) the Junior High School License and 6 (20%) were graduates. Although no statistically significant difference was found, the minimally invasive surgery was associated with the highest socio-economic level.

**Table 1:** Demographic data

	Males, n(%)	Females, n(%)	Mean Age, years (range)	Educational qualification (%)	BMI (range)
Minimally-Invasive	29	1	65,8	13 Elementary School License (43.4)	27,4
Surgery	(96,7%)	(3.3%)	(56-81)	12 Junior High School License (40)	(24,2-30)
				4 graduate (13.3)	
				1 degree (3.3 )	
Open Surgery	28(93,3%)	2(6,7%)	70,3 (48-85)	18 Elementary School License (60)	26,9
				6 Junior High School License (20) 6 graduate (20)	(24,8-29,1)

**Figure 1:**
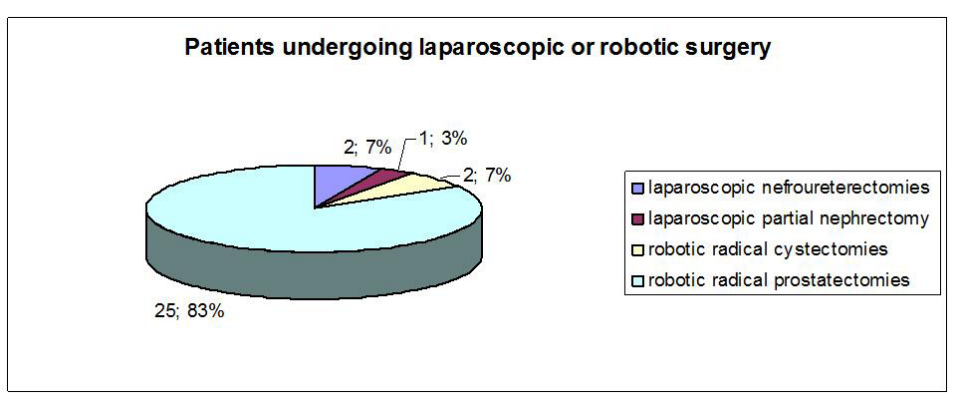
Patients undergoing laparoscopic and robotic surgery

**Figure 2:**
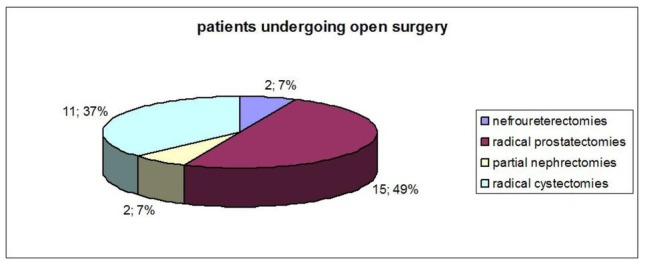
Patients undergoing open surgery

Group 1 included 2 laparoscopic nefroureterectomies (6.7%), 1 laparoscopic partial nephrectomy (3.3%), 2 robotic radical cystectomies with intracorporeal orthotopic ileal neobladder using the Camey II technique (6.7%); 25 robotic radical prostatectomies were taken into consideration (83.3%) (Figure 1); in the second group, we evaluated 2 nefroureterectomies (6.7%), 15 radical prostatectomies (50%), 2 partial nephrectomies (6.7%), and 11 radical cystectomies (36.6%) (Figure 2) with following urinary diversions: 2 ureterocutaneostomies, 5 ileal conduits using the Bricker technique and 4 orthotopic ileal neobladder using the Camey II technique. (Table 2). Superficial incisional SSIs were diagnosed in 10% of cases (3/30): one case in radical prostatectomy and two cases in radical cystectomy with orthotopic ileal neobladder; one patient was treated by close observation, one by oral antibiotics and another by wound incision, drainage and re-suturing. All three cases were Candida Albicans infections. No case occurred in the first group (p <0.05). Space/ organ SSIs developed in 4 patients, which were diagnosed by ultrasound scan and confirmed by abdominal CT: one patient undergone robotic radical prostatectomy (3.3%) showed an infected lymphocele, whereas one case of infected lymphocele after open radical prostatectomy and two cases of pelvic abscess after open radical prostatectomy and radical cystectomy with ileal conduit were detect (10%, p<0.05). All the cases were treated by percutaneous drainage. No urinary tract infection occurred in the two groups. The mean estimated intraoperative blood loss was 233.3 ml (range 100–800 ml) and 493.3 ml (range 200–1200 ml) in group 1 and 2, respectively. Intraoperative bleeding was significantly lower in minimally invasive surgery compared to traditional surgery (p <0.05). During the hospital stay, patients were asked at three different times to define their pain using a number from 0 to 10; the mean value of pain reported by the patients was significantly different between two groups in favor of MIS. That ensures pain reduction, particularly in the early post-operative days (p<0.05): 1.2 vs 4.9 in the first post-operative day, 0.5 vs 3.1 in the third post-operative day; and 0.2 vs 0.4 at the time of discharge, in group 1 and 2, respectively.

**Table 2:** Perioperative outcomes

	Estimated blood loss (range)
Minimally-Invasive Surgery	233.3 ml (100-800)
Open Surgery	493.3 ml (200-1200)

Regarding analgesic treatment, the distribution of the population is quite heterogeneous. In group 1, 7 patients refused analgesic therapy, 12 requested the therapy only for the first post-operative day, 4 for 2 days, 3 for 3 days, 2 for 5 days, and 2 for 7 days. In group 2, 7 patients requested the therapy for the first post-operative day, 4 for 2 days, 5 for 3 days, 2 for 4 days, 2 for 5 days, 1 for 6 days, 2 for 8 days, 1 for 9 days, 1 for 10 days and 1 for 15 days. The mean duration of analgesic treatment was 1.2 days for patients undergoing minimally invasive surgery and 4.2 days for patients undergoing traditional surgery, highlighting a significant difference between the two groups (p <0.05) (Table 2.2).

**Table 2.1:** Mean value of postoperative pain

	Pain (I PostoperativeDay)	Pain (III Postoperative Day)	Pain (Discharge)
Minimally-Invasive Surgery	1.2	0.5	0.2
TraditionalSurgery	3.1	4.9	0.4

**Table 2.2:** Mean duration of analgesic treatment

	Mean Duration of Analgesic Treatment (days)
Minimally-Invasive Surgery	1.2
TraditionalSurgery	4.2

The subjects who underwent MIS responded as very satisfied in 93.3% of cases and quite satisfied in 6.7% of cases; a similar result was obtained for the second group in which 90% were very satisfied and 10% quite satisfied with the received treatment (p> 0.05) (Table 2.3). In MIS group the time to first flatus was shorter (p <0.05) than in open surgery group; indeed, the 83.3% of patients of group 1 experienced initial bowel movement restoration in the first post-operative day, in contrast to those of group 2, who had their bowel movements restored in second post-operative day in 36.7% of cases, and in the third day in 53.3%.

**Table 2.3:** Sample distribution based on satisfaction level

	Very satisfied	Quite satisfied	Quite dissatisfied	Very dissatisfied
Minimally-Invasive Surgery	29	1	0	0
TraditionalSurgery	27	3	0	0

Similarly, MIS was associated with significantly earlier oral diet restoration when compared to open surgery. In the first group, 90% of patients resumed feeding on the first postoperative day; this percentage dropped to 56.7% in the second group (p <0.05). Also, the mobilization occurred significantly earlier in the first group (p <0.05): 90% of patients in group 1 resumed mobilization in the first postoperative day, whereas this percentage declines to 50% in patients of the second group. (Table 2)

The overall complication rate was 20% (6/30) and 66.7% (20/30) in groups I and II, respectively (p <0.05). In group I, there were 3 complications of grade I (10%), 2 of grade II (6.7%) and 1 of grade III-b (3.3%). In Group II, 4 patients presented with grade I (13.3%) and 11 grade II (36.7%) complications; the major complications occurred in 5 patients (16.7%): 3 patients developed a space/organ SSIs (grade III-b), whereas 2 patients who underwent radical cystectomy required resuscitation due to respiratory failure (grade IV-a). The average length of stay in the first group and in the second group was 8.6 days and 11.3 days, respectively. This finding demonstrates that MIS significantly reduces the length of hospital stay (p <0.05) (Table 2.4).

**Table 2.4:** Hospital stay

	Mean hospital stay (range)
Minimally-Invasive Surgery	8.6 days (4-19)
TraditionalSurgery	11.3 days (5-22)

## 4 Discussion

Surgical site infections (SSIs) represent one of the most common complications of surgery; they are associated with prolonged inpatient stay, increased hospital re-admission, mortality, and a detrimental effect on health-related quality of life [16]. In 2006, it was estimated that healthcare-associated infections affected about 8% of hospitalized patients in the UK and that SSIs represented 14% of these infections. [17]. The CDC defines three levels of severity of SSIs:

– superficial incisional, affecting the skin and subcutaneous tissue;
– deep incisional, affecting the fascial and muscle layers;
– organ or space infection, involving any part of the anatomy other than the incision that is opened or manipulated during the surgical procedure [18,19].

The risk factors for developing an SSI are age, comorbidity (American Society of Anesthesiologists score ≥3), diabetes, malnutrition, low serum albumin, radiotherapy and steroid use, high body mass index, host immune status, smoking, site, and level of wound contamination [20]. Further significant risk factors for SSI are related to the type and complexity of the surgical procedure, including duration of operation, emergency surgery, employment of non-reabsorbable suture, extensive electrocautery, massive bleeding, hypothermia, and type of surgical approach (laparotomic or laparoscopic or robotic). Laparoscopic surgery and also robotic surgery were demonstrated to be safe and feasible techniques [21,22].

In this prospective study, we compared the SSI incidence between MIS (laparoscopic and robotic) and open urological surgery. We found a higher rate of superficial incisional SSI in patients undergoing traditional surgery (0% vs 10%, <0.05); similarly, space/organ SSIs were significantly more frequent in open techniques (3.3% vs 10%, p<0,05). No urinary tract infection occurred in either of these two groups. These findings could be explained by different reasons: 1) a smaller surgical incision and the absence of surgeon’s hands into the surgical site may reduce the exposure to potential infection; possibly surgery, an exogenous or endogenous bacterial contamination and proliferation may occur leading to inflammatory reaction of the tissues involved; hence, the inflammatory cells determine the tissue destruction and the formation of pus. Moreover, many local factors, such as the presence of necrotic tissue, hematomas and dead spaces favor bacterial growth and inhibit local tissue resistance [23]. Thanks to more accurate dissection of tissues, lower levels of bleeding and less ischemic suture, MIS allows to reduce the presence of necrotic tissue and hematomas. This leads to less systemic stress, improved immunologic response, and less local tissue trauma.

These factors could be a further reason of lower incidence of SSI in MIS. On the other hand, there are many local factors may play a key role in the development of SSI after open surgery: a longer abdominal wall incision leads to major exposure of tissues to air; the use of retractors, electrocautery and potentially ischemic sutures could devitalize the tissues, resulting in scarring defects. 2) MIS was demonstrated to reduce the blood loss with respect to open surgery: this allows to maintain higher serum levels of albumin and globulin necessary for controlling infection through the immune system; moreover, MIS was associated with a lower rate of transfusion. A recent systematic literature review showed perioperative blood transfusions had an immunosuppressive effect [24,25]. 3) The number of drains and length of catheterization are lower in MIS than in open surgery, reducing thus the risk of infection [26]. However, our findings are consistent with previously published data. Many studies showed lower rate of SSIs in MIS than open surgery [27].

Recently, Tollefson et al. evaluated the incidence of superficial and deep infections of surgical wounds in 5,908 patients undergoing radical prostatectomy by a laparotomic or robotic approach: the authors found that the robotic technique was associated with a lower rate of infections (0.6% vs 4.7%; p = 0.001) [28]. Osmonov et al reported similar results: they found the absence of superficial and deep infections after robotic radical prostatectomy and the only cases of infections; however, organ infections (infected lymphoceles or abscesses pelvic) were significantly lower than after open surgery [29]. In a recent work, Shigemura et al. also compared the postoperative infection between robotic and laparotomic radical prostatectomy; they found a lower ratio of SSI in favor for robotic approach [30]. Finally, Dobson et al demonstrated that SSIs in patients undergoing MIS led to less morbidity than those undergoing an open procedure [31].

The second aim of our study was to compare the peri-operative outcomes of MIS with respect to open surgery. We found that laparoscopic and robotic surgery were associated with lower intraoperative bleeding, less post-operative pain mostly in the early days and consequently less duration of analgesic treatment; also, the time to first flatus, early oral diet restoration, the mobilization and length of hospital stay favored MIS. The overall complication rate was lower in patients undergoing MIS than in those undergoing open surgery (20% vs 66.7%, p<0.05). These findings are consistent with data reported in previous studies. When compared to open surgery, laparoscopic and robotic ones lead to reduction of overall morbidity and better perioperative outcomes [32]. In addition, to obtain a better cosmetic result, MIS was proved to minimize intraoperative bleeding, to reduce postoperative pain, and to allow early mobilization; consequently, the minimally invasive strategy decreased the patient’s discomfort and the length of stay [33, 34, 35, 36, 37].

Although the introduction of robotic technology was not demonstrated to significantly reduce the overall surgical complication rate, many studies showed encouraging results. Radical cystectomy is the gold standard treatment for muscle-invasive bladder cancer and for high risk non muscle-invasive cancers [38]. The laparotomic approach is the standard strategy, whereas laparoscopic and robotic radical cystectomy are still considered to be investigational procedures. However, a recent systematic revision and meta-analysis comparing open vs laparoscopic vs robotic radical cystectomy showed similar intraoperative and a similar 30-day complication rate, whereas grade 3 (based on the Clavien Dindo classification) 90-day complication rate was lower with a robotic technique [39]. These findings were confirmed also by the Pasadena Consensus Panel, a group of experts on radical cystectomy, lymphadenectomy, and urinary diversion [40]. Also, based on our results MIS was associated with lower risk of complications: a statistically difference of overall complication rate was found between open and MIS, although there was no difference concerning major complications. This finding was probably due to the small number of major complications in both groups. The main limitation of the study was the small sample size.

## 5 Conclusions

The use of minimally invasive techniques in urological surgery reduced the risk of SSI by comparison with a traditional approach. In addition, MIS was associated with better peri-operative outcomes and a lower overall complication rate. These observations should be considered in decision-making concerning the surgical strategy to be used.
